# User-Information-Aware D2D Multicast File Distribution Mechanism

**DOI:** 10.3390/s18103389

**Published:** 2018-10-10

**Authors:** Hongcheng Huang, Biao Liu, Min Hu, Yang Tao, Wei Xiang

**Affiliations:** 1 School of Communication and Information Engineering, Chongqing University of Posts and Telecommunications, Chongqing 400065, China; s160131095@stu.cqupt.edu.cn (B.L.); humin@cqupt.edu.cn (M.H.); s170131014@stu.cqupt.edu.cn (W.X.); 2 Chongqing Engineering Research Center of Communication Software, Chongqing 400065, China

**Keywords:** D2D, multicast communication, content distribution, user information aware, social aware

## Abstract

There are a large number of redundant transmissions in current D2D multicast content delivery systems, which seriously reduces the utilization efficiency of resources. This paper designs a novel user-information-aware D2D video distribution mechanism. More specifically, by predicting users’ video requests, the video can be pushed to potential service requesters while distributing video for service requesters. Firstly, the willingness of potential requesters to accept the pushed video is estimated based on the users’ interests, the popularity of the videos and the residual-energy of the users’ devices, and the user-demand-aware clustering algorithm is proposed. Secondly, considering social and interference information, the utility metric of D2D multicast is proposed to measure the value of content distribution service. Finally, this paper proposes a D2D video distribution mechanism to optimize the utility value. Simulation results show that the proposed mechanism significantly improves throughput, energy and spectrum efficiency compared to the traditional distribution mechanism.

## 1. Introduction

With the rapid development of Mobile Internet, it is estimated that mobile data traffic will increase by approximately seven times from 2016 to 2021, in which 78% of mobile data traffic is generated by mobile video services [1,2]. The explosive growth of data traffic will put tremendous pressure on existing mobile communication systems, and D2D communication is recognized as a key technology to solve this problem. D2D communication allows direct data communication between adjacent terminals without the assistance of base stations, which has great potential for alleviating the pressure on base stations, increasing system capacity, enhancing network coverage, and increasing transmission rate [3,4]. In fact, there is greater redundancy in the transmission of multimedia data such as video and music in the mobile communication system, seriously reducing the efficiency of resource utilization [5]. In order to reduce the redundant data transmission and further improve the system capacity and resources utilization, the use of D2D multicast communication to distribute multimedia content to users has been received widespread attentions and researches in recent years [6,7,8].

Cooperative communication is an efficient D2D communication paradigm where devices can serve as relays for each other [9]. The relay-assisted D2D communication occupies a major position in the research of existing D2D multicast communication. The D2D multicast cluster is formed by nearby users with the same service demands, and the cluster head node as relay node selected by base stations assists other nodes in communication. The content requested by the members of the D2D multicast cluster is sent to the cluster head, and then cluster head distributes content to other cluster members by D2D multicast communication. Recently, the relay-assisted D2D communication has been considered in the standardization process of mobile broadband communication systems. For instance, it is proposed in the 3rd Generation Partnership Project (3GPP) Long Term Evolution Advanced (LTE-Advanced) to improve the communication quality of cell edge users.

Based on the relay-assisted D2D communication and D2D multicast communication, the redundant transmission of multimedia content distribution can be reduced greatly, dramatically improving the efficiency of spectrum and energy, and further mitigating the shortage of spectrum resources. However, the relay node and cluster head node always cause excessive energy consumption during assisting content distribution and its resources also be occupied. The selfishness of the users may cause relay nodes to behave negatively in assisting D2D communication. The user’s mobility and social relationships among users can also seriously affect the data transmission performance [10,11]. What’s more, the performance of D2D multicast communication is limited by the worst interference plus noise ratio (SINR). Therefore, formulating a suitable clustering mechanism and selecting the appropriate user as the relay node or cluster head are the key to improve the performance of D2D multicast communication [12,13]. Currently, relevant research in this field is very active and has achieved fruitful research results. However, by tracking the behavior of users accessing video, Li et al. [14] found that the amount of video access during the daytime was relatively stable, although the amount of video accessed by users during various hours varied greatly. In other words, videos are heavily accessed by users at any time during the day. For the high popularity videos, the same video in the same area should be transmitted multiple times at different times by using existing D2D multicast communication technology, so there is still much space to improve energy efficiency and spectrum utilization. In order to solve the above problem and further reduce the redundant transmission, the user-information-aware D2D multicast file distribution mechanism is designed in this paper, and then the corresponding clustering and cluster head selection algorithms are proposed. The main contributions of this work are summarized as follows.

- This paper proposes a user-information-aware D2D multicast file distribution mechanism, the video is pushed to potential service requester when distributing video for service requesters, the energy and spectrum efficiency can been improved dramatically by increasing the number of D2D multicast receivers.
- To guarantee the quality of experience (QoE) of potential service requesters, the willingness of potential requesters to accept the pushed video was calculated by considering users’ interest and the status information about user’s device, and then a clustering algorithm based on the user’s willingness is developed.
- The utility metric of D2D multicast communication is proposed to evaluate the D2D multicast content distribution service. Meanwhile, the link quality information and social relationships among users are taken into the calculation in the utility value to suit the actual communication environment.
- A cluster head selection algorithm is developed to maximize the utility value of D2D multicast communication.

The rest of this paper is organized as follows: the related works are introduced in Section 2. The system model is given in Section 3. A user-demand-aware method and a clustering algorithm based on the user’s willingness are proposed in Section 4. The social information awareness method is introduced in Section 5. The cluster head selection algorithm that maximizes the utility value of D2D multicast communication is proposed in Section 6. Simulation results are analyzed in Section 7. Lastly, the conclusions are given in Section 8.

## 2. Related Works

In the traditional cellular communication system, an enormous number of users with the same video demands separately obtain the required content from the base station using their respective allocated bandwidth, so the base station needs to send duplicate content over different links as the multiple users initiate the same service request. Obviously, the base station performs a vast number of redundant transmissions, resulting in low video transmission efficiency, and the amount of redundancy is proportional to the popularity of the transmitted content. D2D multicast content distribution as a potential technology to reduce redundant transmissions and improve the resource utilization, has received extensive attention and research in recent years. As mentioned in Section 1, the improvement on performance in D2D multicast communication is influenced by selecting suitable clustering, cluster head selection methods and data distribution mechanisms.

In the D2D multicast communication, the cluster head node is responsible for relaying data, so the transmission performance of the content distribution system is mainly determined by the capability of the cluster head node. And the capability of cluster head includes two aspects, physical and social. In order for all receivers to receive all data correctly, the transmission rate of D2D multicast communication is determined by the receiver with the worst channel quality, i.e., the multicast communication performance is subject to the worst SINR of the receivers. In [15], with consideration of the difference of link quality, a SINR constraint-based intra-cloud D2D multicast retransmission algorithm is proposed. The link cost model is built in the proposed algorithm, limiting the number of multicast retransmissions, and selecting the appropriate cluster head by traversing the link quality matrix. The proposed algorithm reduces the link consumption, and the spectrum efficiency can be improved. The efficient Tx user selection algorithm is proposed in [16], in which the number of stable links for each node in the cluster is calculated by the link quality information about nodes, and then the user with the largest number of stable links is selected as the cluster head. The throughput of D2D multicast communication have improved by maximizing the number of members in the cluster. This algorithm reduces interference in D2D multicast transmissions, increasing the throughput of more than 5% of edge users by at least 50%.

The above studies only consider the physical information. In fact, numerous studies have confirmed that the social relationship between users has a significant impact on the performance of wireless communications [17,18]. Cluster head nodes forward data for other users in the system, so cluster head nodes are frequently referred to as relay nodes. The relay nodes will cause a large amount of storage, calculation, and energy burden as providing services for other users. In addition, different users have various file or video demands, so it is very likely that the relay nodes will cooperate negatively. The stability of D2D links can be affected by the users’ selfishness, mobility and degree of intimacy between users. Insufficient intensity of social relationships between users can even lead to frequent breaks in D2D links, severely reducing the ability in D2D communication and the user’s experience quality. Therefore, it is necessary to integrate social domain information and social network research methods into the research of D2D multicast communication.

The social-aware file-sharing mechanism is proposed in [19], users with the same documentation requirements are divided into the same D2D multicast cluster, and then the cluster is selected by the social relationships and trust degrees of users. In the proposed mechanism, the influence of users’ social relationships to wireless communication is fully considered, user’s experience quality has improved greatly, and the transmission time delay and the burden of base station have reduced. In [20], the notion of social-aware rate is proposed by combining the social selfishness with the link rate, which can ensure the physical link quality and effective collaboration between users, and then the problem of social-aware-rate-based file sharing mode selection is modeled as a maximum weight mixed matching problem. In order to eliminate the negative impacts of relay node’s selfishness, the user’s interest factor is considered into the establishment of D2D file sharing cluster in [21], the proposed cosine-similarity-based greedy intra-cluster caching scheme has achieved near-optimal delay performance. In [22], a trust-oriented partner selection mechanism (TPSM) is proposed, which can avoid choosing those users with non-cooperative behavior as cooperative users. With the consideration of user’s psychological structure, the multi-dimensional trust relationships between sending users and cooperative users is built, including cognition, emotion, and behavior. In addition, the cooperative nodes are stimulated to actively participate in data distribution so as to further eliminate the negative impact of user selfishness. In [23], a Stackelberg game-based pricing mechanism is proposed to inspire the core users to assist in the distribution of videos. The proposed mechanism provides guarantees for the benefits of the system and the core users, improving the effectiveness and reliability of the system. Integrating social domain information into the traditional D2D multicast communication research method is closer the actual communication environment, further improving the D2D multicast communication performance.

The relay nodes can consume a large amount of energy when forwarding data for other users, but the battery capacity of relay nodes is limited. Therefore, in the relevant research areas, besides the efforts to improve the data distribution rate, delay performance, and system capacity, there are a large number of domestic and international researches which devoted to optimizing the energy efficiency of the system [24,25,26]. To cope with the increasing demand for local services in 5G cellular networks, literature [27] proposed a new cellular communication architecture that integrates energy harvesting technology and social networking features into D2D communication for local data dissemination. The proposed architecture includes three domains: the physical domain, the energy domain, and the social domain, which enables a significant improvement in the spectrum efficiency and energy efficiency of localized data transmission. A joint power and resource allocations scheme is proposed in [28] with a goal of improving the energy efficiency, maximizing the number of accessed D2D groups, and minimizing the total terminal transmission power for D2D underlay multicast communication at the same time. 

In order to improve the performance of D2D multicast data distribution such as throughput, spectrum efficiency, and energy efficiency, the above studies have proposed their own solutions. Nonetheless, most videos are accessed by a large number of users at any time during the day, so the same videos in the same area are transmitted multiple times at different times by using existing D2D multicast communication technology, bring about a large amount of redundant transmissions. To tackle the aforementioned redundant transmission and improve the resource utilization efficiency of the D2D multicast communication system, this paper proposed a novel user-information-aware D2D multicast file distribution mechanism, in which the video is pushed to potential service requesters when distributing video for requesters. The throughput has been improved greatly by increasing the number of D2D multicast receivers, and in turn the energy efficiency and spectrum efficiency have been improved dramatically. 

## 3. System Model

### 3.1. Problem Description

In the cell, the members of the D2D multicast cluster are composed of the geographically adjacent users that request the same video from the base station, and the cluster head is selected from the members of the cluster. The base station firstly sends the requested video to the cluster head through the cellular downlink, and then the cluster head distributes the video to other members via D2D multicast communication. In this way, the redundant transmission can be reduced for the same video, improving the utilization of the spectrum and offloading the traffic of the base station.

Assuming that the D2D link reuses the uplink channel resource of a cellular user in the same cell, let $P_{h}$ denotes the D2D transmit power of cluster head. As mentioned in the above, the performance of D2D multicast communication is limited to the worst SINR of receivers. *SINR*^min^ is the worst SINR of receivers, *u*_m_ denotes the receiver with the worst SINR, and then *SINR*^min^ can be denoted as:

(1) $$SINR^{\min}=\frac{p_{h}g_{hm}}{n_{0}+I_{cm}}$$

where $n_{0}$ is the noise power of the AWGN model, $I_{\mathrm{cm}}$ is the interference signal power of user *u_m_* received from the cellular user, and $g_{\mathrm{hm}}$ is the channel gain between the cluster head user and user *u*_m_. Therefore, according to Shannon Theorem, the channel capacity of the D2D multicast communication can be expressed as:

(2) $$C_{total}=NB\log_{2}(1+SINR^{\min})$$

where *N* is the number of receivers in the D2D multicast cluster, *B* is the bandwidth. Observing (1) and (2), we know the throughput can be greatly improved by increasing the number of members in the D2D multicast cluster in certain conditions, dramatically promoting the energy and spectrum efficiency.

### 3.2. System Model

In the existing D2D multicast communication system, the time domain is not fully considered in distribution for user service requests. Actually, most of the users access the video at any time during the day. For the high popularity videos, the same video in the same area more likely be transmitted multiple times at different times by using existing D2D multicast communication technology. Therefore, there are a large number of redundant transmissions of the traditional D2D multicast content distribution system, the spectrum efficiency and energy efficiency of the system still can be improved.

**Figure 1:**
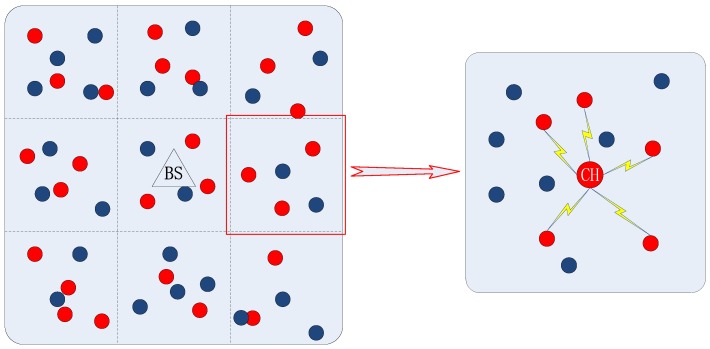
Grid-based clustering method and the D2D multicast scene in the grid.

The users requesting video at the current moment are called service requesters, and the potential service requesters are defined as the users presenting the same video demands with service requesters but no initiate service requests at this moment. The distribution of users within the cell is shown as Figure 1, where the blue dots and red dots represent the requesters and the potential requesters, respectively. The cell is divided into the grid based on the grid-based clustering deduced in [23]. In the same grid, the users needing the video distribution service at this moment form the D2D multicast cluster, so the distance between every two users should be less than the D2D communication radius.

Figure 1 also shows the distribution of users within a grid and the D2D multicast scene in the grid. In this grid, there are *n* service requesters and *m* potential service requesters, denoted by the set $U_{R}=\left\{u_{r1}{,u}_{r2}{,\ldots ,u}_{\mathrm{rn}}\right\}$ and the set $U_{P}=\left\{u_{p1}{,u}_{p2}{,\ldots ,u}_{\mathrm{pm}}\right\},$ respectively. The users set in the grid is constituted by requesters and potential requesters, denoted by the set ${U=U}_{R}\cup U_{P}$. In the traditional D2D multicast system, the members of the D2D multicast cluster are formed by service requesters, and an advisable user is selected as the cluster head from the service requesting users. However, the traditional mechanism fails to give full play to the advantages of D2D multicast communication, and the performance of the system can be further promoted. 

If the video is pushed to the potential service requesters when it is distributed to the service requesters, the throughput of the system will be significantly improved due to increased number of D2D multicast receivers, thereby the energy efficiency and spectrum efficiency will be improved greatly. Besides, the QoE of potential service requesters will be also improved by the suitable video push service. In fact, due to the differences of user’s interest, the residual energy of user’s device, and the status of user’s device, the willingness of each potential requesters to accept the pushed video is very different. What’s more, the channel quality and the social relationship strength among users also affect the transmission performance of the system. Therefore, the selection of both potential service requesters and the cluster head is the key to improve the transmission performance of D2D multicast file distribution mechanism. The purpose of this paper is to accurately predict the service requests of potential service requesters and formulate appropriate clustering and cluster head selection schemes to achieve system throughput, energy efficiency and spectrum efficiency improvement.

## 4. User’s Demands Awareness

The potential service requesters receiving the pushed video will consume the energy of the user’s device, occupying the device’s bandwidth and storage space. If the device’s residual energy or storage space is insufficient, or the potential service requester is using the wireless network, the user may be unwilling to accept the pushed video. In addition, the user’s subjective intention is also an important factor to affect the video push service. If the pushed video does not meet the user’s interest, the push will lose its significance, and the user’s experience quality will be seriously reduced. In order to accurately to predict user’s service requests and improve the user’s experience quality, the willingness of potential service requester to accept the pushed video is studied in this section, and then the user-demand-aware clustering algorithm is proposed.

### 4.1. Video Request Probability

A certain video requested by users is mainly related to the user’s interest and the video’s popularity. Therefore, based on the user’s interests and video’s popularity, the calculation of video request probabilities can take into account the commonness of the video and the user’s personality simultaneously.

#### 4.1.1. User’s Interest

The user’s interest can be reflected by the accumulated duration information accessing video content to some extent. The larger watching times clearly indicate the higher interest degree for a certain interested video. Thus, the user’s interest in the video can be shown in (3):

(3) $$p_{i,k}=\frac{t_{i,k}}{t_{i}}$$

where $p_{i,k}$ denotes the interest of user $u_{i}$ about the *k*-th type of video, $t_{i,k}$ is the total time of user $u_{i}$ watching the *k*-th type of video, and $t_{i}$ is the total time of user $u_{i}$ watching all videos.

In fact, the duration of different videos is very different, i.e. between over two hours for movies and a few minutes for a small video, so user’s degree of interest just expressed by the ratio of viewing time is not accurate. The proportion, the number requesting this video to the total video requests, should be considered in the calculation of the user’s interest degree. Let $f_{i,k}$ denotes the ratio between the number of times user $u_{i}$ watching the *k*-th type video and the total video views in the past period of time *T*, it can be expressed as:

(4) $$f_{i,k}=\frac{x_{i,k}}{X_{i}}$$

where, $x_{i,k}$ denotes the number of times user $u_{i}$ watching the *k*-th type video, and *X_i_* indicates the total number of videos user $u_{i}$ has watched in the past period of time *T*. The user’s interest can be measured more accurately by simultaneously considering the frequency information and duration information about the user watching the online videos.

In addition, the calculation of the user’s interest degree may be influenced by some interference information generated by the user’s inadvertent touch. Therefore, based on filtered this part of information, let $P_{i,k}^{\mathrm{interest}}$ denotes the interest degree of user $u_{i}$ about the *k*-th type video, and it can be given by:

(5) $$P_{i,k}^{interest}=\delta \frac{x_{i,k}}{X_{i}}+(1-\delta )\frac{\sum_{j=1}^{x_{i,k}}t_{i,k}^{j}}{t_{i}}$$

where, δ is the weight coefficient, $t_{i,k}^{j}$ denotes the duration of user $u_{i}$ *j*-th watching the *k*-th type video. If δ > 0.5, the frequency information has greater influence on the interest degree, so user’s random browsing may cause a large error in the calculation of the degree of interest. If δ < 0.5, the duration information has greater influence on the interest degree, long videos have a great impact on the accuracy of the calculations of interest degree. Notably, each video viewing information in the Equation (5) should satisfy the constraint condition $t_{i,k}^{j}>t_{\mathrm{th}}$, $t_{\mathrm{th}}$ is the time threshold for judging whether a mistaken touch has occurred, namely, it is deemed to inadvertent touch when the time duration watching the video is less than the threshold, and this video viewing information will not be considered in the calculation of the user’s degree of interest.

#### 4.1.2. Video Popularity

Tracking the video viewing rankings of YouTube websites in different regions it was found that the popularity of videos in specific regions follows the Zipf distribution, although the popularity in different regions is different [29]. Let *F* denotes the set of videos requested by the users in the cell over the past period of time *T*, |F| is the number of videos, so the popularity of video $f_{l}$ ranked *l* can be expressed as:

(6) $$P_{l}^{pop}=\frac{l^{-\gamma }}{\sum_{c=1}^{\left|F\right|}c^{-\gamma }},1\leq l\leq |F|$$

where, γ is the Zipf distribution index in the region, $P_{l}^{\mathrm{pop}}$ is the popularity of video $f_{l}$.

The probability that a user requests a certain video is mainly determined by the user’s interest in the video and the popularity of the video. Let’s assume that the video $f_{l}$ requested by the service requester is classified the category *k*. The $P_{\mathrm{pi},l}^{\mathrm{interest}}$ denotes the degree of interest of potential requester $u_{\mathrm{pi}}$ in video $f_{l}$, and then the degree of interest $P_{\mathrm{pi},l}^{\mathrm{interest}}$ can be calculated according to Equation (5). The probability that potential requester will request video $f_{l}$ later can be expressed as:

(7) $$P_{pi,l}^{request}=\lambda P_{l}^{pop}+(1-\lambda )P_{pi,l}^{interest}=\lambda \frac{l^{-\gamma }}{\sum_{c=1}^{\left|F\right|}c^{-\gamma }}+(1-\lambda )\left[\delta \frac{x_{pi,k}}{X_{pi}}+(1-\delta )\frac{\sum_{j=1}^{x_{pi,k}}t_{pi,k}^{j}}{t_{pi}}\right]$$

where λ is the weight coefficient, *k* is the category number of video $f_{l}$, and $P_{\mathrm{pi},l}^{\mathrm{interest}}\in$ [0,1], where the above equation must satisfy the constraint conditions $t_{\mathrm{pi},k}^{j}>t_{\mathrm{th}}$ and 1≤l≤|F|.

### 4.2. User’s Willingness

In contrast to the transmitter, the energy consumption when receiving the video file mainly comes from its own power use. Therefore, according to the radio energy dissipation model proposed in [30], the energy consumption receiving *n* bits for a mobile terminal is given by:

(8) $$E_{r}(n)=nE_{elec}$$

where, $E_{\mathrm{elec}}$ denotes the circuit loss constant. Let $E_{\mathrm{pi}}$ denote the total energy that the potential service requester’s device can store, $E_{\mathrm{pi}}^{s}$ denotes the current residual energy of the device for potential service requester $u_{\mathrm{pi}}$, and the file size of video $f_{l}$ is $n_{l}$ bits. In order to ensure the potential requester’s QoE, the device’s residual energy ratio $\varphi_{pi}$ for user $u_{\mathrm{pi}}$ should be greater than the threshold $\varphi_{th}$ after the video is completely received, which can be expressed as:

(9) $$\varphi_{pi}=\frac{E_{pi}^{s}-n_{l}E_{elec}}{E_{pi}}>\varphi_{th}$$

where the value of $\varphi_{th}$ has been indicated in [31], i.e., $\varphi_{th}$ = 0.3. If the ratio of the residual energy of the user’s device after receiving the pushed video is lower than the threshold $\varphi_{th}$, the potential requester will not accept the video push service. Therefore, the expression of the lowest residual energy coefficient *c* is expressed as:

(10) $$c=\left\{ \begin{array}{ll} 1 & \varphi_{pi}>\varphi_{th}\\ 0 & \varphi_{pi}\leq \varphi_{th} \end{array}\right.$$

In summary, when cluster head attempt to improve throughput by pushing video $f_{l}$ to potential requester $u_{\mathrm{pi}}$, the willingness of user $u_{\mathrm{pi}}$ to accept the pushed video $f_{l}$ can be expressed as:

(11) $$P_{pi,l}^{accept}=cP_{pi,l}^{request}=\left\{ \begin{array}{ll} \lambda P_{l}^{pop}+(1-\lambda )P_{pi,l}^{interest} & \varphi_{pi}>\varphi_{th}\\ 0 & \varphi_{pi}\leq \varphi_{th} \end{array}\right.$$

### 4.3. User-Demand-Aware Clustering Algorithm

As mentioned above, the probability of the user requesting a video is calculated according to the historical viewing behavior information of the user and the video’s popularity. Besides, the extra reception of the pushed video will consume the energy of the user’s device, occupying the device’s bandwidth and storage space. In order to ensure the experience quality of the potential service requesters, and avoid disturbing other unrelated users with the video push service., the probability of accepting the pushed video, named as user’s willingness $P_{\mathrm{pi},l}^{\mathrm{accept}}$, is calculated according to the status information of the user’s device, the popularity of the pushed video and user’s degree of interest. In the construction of the D2D multicast cluster, with the consideration of user’s willingness $P_{\mathrm{pi},l}^{\mathrm{accept}}$, any potential service requester $u_{\mathrm{pi}}$ who is selected as a push object to accept the video $f_{l}\in F$ should meet the following conditions:

(12) $$P_{pi,l}^{accept}>P_{th}^{accept},\forall u_{pi}\in U_{P}^{S}$$

where, $U_{P}^{S}$ denotes the set of potential service requesters who are selected as push objects, and $U_{P}^{S}\subseteq U_{P}$. $P_{\mathrm{th}}^{\mathrm{accept}}$ is the minimum willingness threshold for potential service requesters to accept the pushed video, meaning that the user’s QoE may be seriously degraded when the user’s willingness $P_{\mathrm{pi},l}^{\mathrm{accept}}$ accepting the pushed video is less than the threshold $P_{\mathrm{th}}^{\mathrm{accept}}$. Besides, the willingness value is considered to 1 when the video requested by the service requesters. 

Taken together, when the requesters initiate a request for the video $f_{l}\in F$, the system constructs a D2D multicast cluster by added requesters and potential requesters fitting (12). Therefore, the users set $U_{C}$ of the D2D multicast cluster can be expressed as:

(13) $$U_{C}=U_{R}\cup U_{P}^{S}$$

According to the above analysis, this paper proposes a novel user-demand-aware D2D multicast clustering algorithm, which is based on the grid-based clustering algorithm, as shown in Algorithm 1. First, the willingness of the potential requester to accept the pushed video is calculated, and then the user set $U_{C}$ of the D2D multicast cluster is composed of the requesters and the potential requesters who satisfy the condition of the willingness threshold. In the proposed algorithm, the throughput and resource utilization efficiency will be improved greatly by increasing the number of D2D multicast receivers. The willingness value for the service requester is 1, so they are directly put into the user set $U_{C}$ of D2D multicast cluster when the algorithm starts.


**Algorithm 1. User-Demand-Aware Clustering Algorithm**

**1.**

**Input:**

**2.**
service requesters set $U_{R}$, potential service requesters set $U_{P}$, the popularity of
video $f_{l}\in F$, user $u_{\mathrm{pi}}\in U_{P}$ and their device’s status information
**3.**

**Output:**

**4.**
cluster members set $U_{C}$

**5.**

**Procedure User Demand-aware Clustering Algorithm**

**6.**

**for**

$u_{i}\in U_{R}$

**do**

**7.**
    put $u_{i}$ into set $U_{C}$
**8.**

**end for**

**9.**
**for** $u_{\mathrm{pi}}\in U_{P}$**do**
**10.**
    calculate the willingness $P_{\mathrm{pi},l}^{\mathrm{accept}}$ of user $u_{\mathrm{pi}}$ to accept video push service
    according to Equations (5)(6)(9)(11)
**11.**
    **if**
$P_{\mathrm{pi},l}^{\mathrm{accept}}>P_{\mathrm{th}}^{\mathrm{accept}}$
**then**
**12.**
         put $u_{\mathrm{pi}}$ into set $U_{C}$

**13.**
    **end if**
**14.**

**end for**

**15.**

**End**


## 5. Social Relationship Awareness

**Figure 2:**
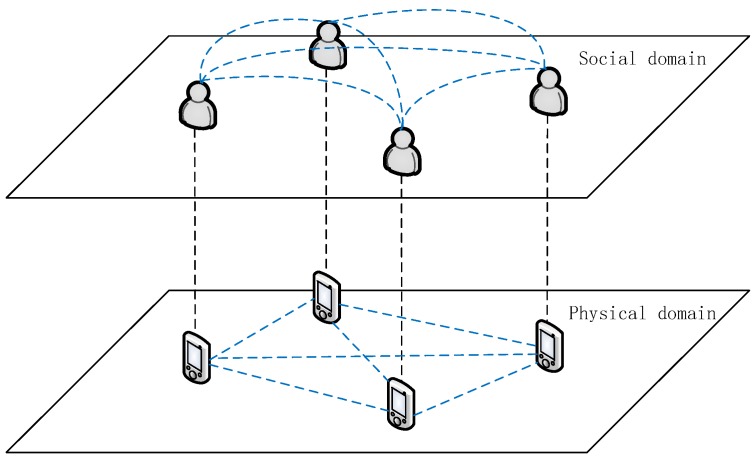
The relationships of D2D communication devices in social domain and physical domain.

Due to the mobility of D2D users, the limited transmission capability for wireless terminal devices and the instability of wireless channels, the optimal performance of D2D communication cannot be achieved in the actual D2D communication environment, and even D2D links may be frequently broken, so the stability of the D2D link is vital to whether the D2D communication system can achieve long-term stable performance gain. In addition to the aforementioned physical domain factors, recently, the numerous studies show that the performance of D2D communication is closely related to the social relationships between users. The strength of social relationship can be reflected by the attributes of social relationship such as the degree of trust and the demand similarity. The greater the strength of social relationships, the stronger the social stability between users. The relationships between D2D communication devices in social domain and physical domain are shown in Figure 2, and a stable D2D communication link can be established only when both the social and the physical link have strong stability.

In the same grid, users are likely to request the same video file if users have similar video viewing demands, even receiving files in the same D2D multicast cluster. That’s to say, the strength of social relationships can be expressed by the demand similarity between users, and the demand similarity can be calculated from the video request probability. In the Equation (7), the probability of any user $u_{i}\in U_{C}$ requesting video file $f_{l}\in F$ can be calculated by:

(14) $$P_{i,l}^{request}=\lambda P_{l}^{pop}+(1-\lambda )P_{i,l}^{interest}$$

According to (14), the probability vector of user $u_{i}\in U_{C}$ requesting videos can be expressed as:

(15) $$P_{i}^{request}=\left(\begin{array}{ccc} P_{i,1}^{request} & \ldots & P_{i,|F|}^{request} \end{array}\right)$$

The probability vector $P_{i}^{\mathrm{request}}$ can reflect the video viewing demands of user $u_{i}\in U_{C}$. Where |F| is the number of videos in the set *F*. As mentioned, the strength of social relationships can be evaluated by the user’s demand similarity, which is more realistic than simply using user’s interest similarity. Therefore, the strength of social relationship between user $u_{i}$ and user $u_{j}$ can be calculated by:

(16) $$s_{i,j}=\frac{P_{i}^{request}\cdot P_{j}^{request}}{\left||P_{i}^{request}||||P_{j}^{request}|\right|}=\frac{\sum_{l}^{\left|F\right|}P_{i,l}^{request}P_{j,l}^{request}}{\sqrt{\sum_{l}^{\left|F\right|}{\left(P_{i,l}^{request}\right)}^{2}}\times \sqrt{\sum_{l}^{\left|F\right|}{\left(P_{j,l}^{request}\right)}^{2}}}$$

Due to the non-negative probability, the product of the probability vectors requested by user $u_{i}$ and user $u_{j}$ is also non-negative, namely $s_{i,j}\in$ [0,1]. According to Equation (16), the social relationship strength matrix **S** about set $U_{C}$ can be expressed as:

(17) $$S=\left[\begin{array}{ccc} s_{1,1} & \ldots & s_{1,|U_{C}|}\\ \ldots & \ldots & \ldots \\ s_{|U_{C}|,1} & & s_{\left|U_{C}|,|U_{C}\right|} \end{array}\right]\begin{array}{c} \left|U_{C}|\times |U_{C}\right| \end{array}$$

where, ${|U}_{C}|$ is the number of users in the set $U_{C}$, and if i=j, then $s_{i,j}=1$.

## 6. Cluster Head Selection Mechanism

In the fourth section, the probability of the user requesting the video and the willingness for the potential service requester to accept the pushed video were deduced by analyzing the user’s historical viewing information, the popularity of the video and the state information about the user’s device. Using the user-demand-aware D2D multicast clustering algorithm, the potential service requesters that satisfy the condition of the willingness threshold were selected to form a D2D multicast cluster together with the service requesters. In D2D multicast communication, the selection of cluster head has a great influence on the performance of data transmission. Therefore, in this section, we propose a D2D multicast utility metric to measure the transmission performance of D2D multicast communication, and then a cluster head selection algorithm is proposed to optimize the system’s D2D multicast utility value.

### 6.1. D2D Multicast Utility Metric

**Figure 3:**
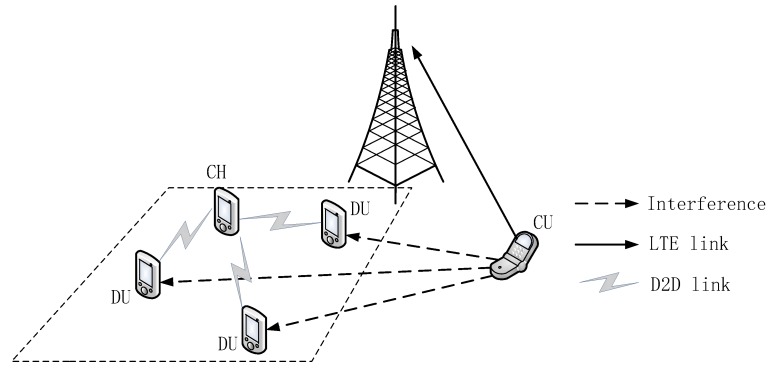
D2D multicast communication interference diagram.

As shown in Figure 1, several service requesters and potential service requesters are randomly distributed in the grid. In the fourth section, with consideration of the user’s willingness and other information, the potential service requesters are selected to form the users set $U_{C}$ of D2D multicast cluster together with the service requesters. As shown in Figure 3, CU denotes the cellular user, DU denotes D2D user, and CH denotes the cluster head of the D2D multicast cluster. D2D users can reuse the uplink channel resource with cellular user, so the D2D multicast receivers will be disturbed by cellular user’s transmit signals. In this paper, the channel model follows path fading and Rayleigh fading, each channel obeys one-mean Rayleigh fading independently [31]. When the cluster head CH and cellular user CU transmit signals with constant power $P_{h}$ and $P_{c}$ respectively, the SINR for any D2D multicast receiver $u_{i}$ in the set $U_{C}$ can be expressed as:

(18) $$SINR_{h,i}=\frac{P_{h}d_{h,i}^{-\alpha }h_{0}}{P_{c}d_{c,i}^{-\alpha }h_{0}+\sigma^{2}}$$

where, $d_{h,i}$ is the distance between the cluster head CH and the D2D multicast receiver $u_{i}$, $d_{c,i}$ is the distance between the cellular user CU and the D2D multicast receiver $u_{i}$, α is the path loss exponent, $h_{0}$ is the Gaussian channel coefficient constant, $\sigma^{2}$ is zero-mean the Gaussian noise. The CH and $u_{i}$ are members of the user set $U_{C}$, and each user in the set $U_{C}$ may be selected as cluster head. Due to the differences of channel quality for users, the received SINR of D2D multicast users have very differences when select different user as cluster head. Assuming that the relative distance between all users in set $U_{C}$ are known by the system, and let **D** denotes the distance matrix about the user set $U_{C}$, it can be expressed as:

(19) $$D=\left[\begin{array}{ccc} d_{1,1} & \ldots & d_{1,|U_{C}|}\\ \ldots & \ldots & \ldots \\ d_{|U_{C}|,1} & \ldots & d_{\left|U_{C}|,|U_{C}\right|} \end{array}\right]\begin{array}{c} \left|U_{C}|\times |U_{C}\right| \end{array}$$

Notably, if i=j, then $d_{i,j}=0$. According to Equations (18) and (19), when user $u_{i}\in U_{C}$ is selected as cluster head, and its transmission power is $P_{h}$, the SINR for each D2D multicast receiver can be calculated easily. Thereby, the channel quality matrix about the user set $U_{C}$ can be denoted by:

(20) $$\Gamma_{sinr}=\left[\begin{array}{ccc} \infty & \ldots & SINR_{1,|U_{C}|}\\ \ldots & \ldots & \ldots \\ SINR_{|U_{C}|,1} & \ldots & \infty \end{array}\right]\begin{array}{c} \left|U_{C}|\times |U_{C}\right| \end{array}$$

where ${SINR}_{i,j}$ is the receive signal to interference and noise ratio of user $u_{j}$ when user $u_{i}$ is selected as cluster head. Obviously, if i=j, then ${SINR}_{i,j}=\infty$, because the transmission distance is 0, the signal power does not attenuate.

According to Equation (2), the total throughput of D2D multicast communication is related to the number of D2D multicast receivers and the worst receive signal to interference and noise ratio. The number of D2D multicast receivers is ${|U}_{C}|-1$, and the worst receive signal to interference and noise ratio ${SINR}_{i}^{min}$ can be achieved easily by traversing the channel quality matrix $\Gamma_{sinr}$. The ${SINR}_{i}^{min}$ is the receive signal to interference and noise ratio with the lowest channel quality’s user in the set $U_{C}$ when user $u_{i}$ is selected as cluster head. Therefore, when selected user $u_{i}$ as cluster head, the total throughput of D2D multicast communication can be calculated as:

(21) $$C_{i}=B\left(|U_{C}|-1\right)\log_{2}\left(1+SINR_{i}^{min}\right)$$

where *B* is the channel bandwidth.

In fact, the throughput model shown in Equation (21) ignores the transmission performance influence by the strength of social relationships among users. As mentioned, social relationships among users, which can be described the different levels of trust, familiarity, and demand similarity among users and so on, affect the stability of D2D links. In order to accurately measure the transmission performance of the D2D multicast communication system, the D2D multicast utility metric $C_{i}^{e}$ is proposed in this paper, as shown in (22):

(22) $$C_{i}^{e}=B\left(\sum_{j\neq i}^{\left|U_{C}\right|}s_{i,j}\right)\log_{2}\left(1+SINR_{i}^{min}\right)$$

where $s_{i,j}$ is the strength of social relationship between user $u_{i}$ and user $u_{j}$, its value can be obtained from the social relationship strength matrix **S**. The D2D multicast utility metric $C_{i}^{e}$ reflects the average throughput that the system can achieve when considering the instability of the D2D links. In the calculation of utility metric $C_{i}^{e}$, both the social relationship strength information and channel quality information between users are taken into account, which makes it closer to the actual communication environment than the traditional throughput model.

### 6.2. Utility Optimal Cluster Head Selection Algorithm

The D2D multicast utility metric, an evaluation metric for D2D multicast transmission performance, reflects certain information such as D2D multicast transmission rate and system throughput. From Equation (22), the value of D2D multicast utility metric is mainly determined by the strength of social relationships and channel quality between the cluster head and other members. Due to the different social relationships among users, each user shows different degrees of social activity, namely some users have poor social relationships with other members, while others have strong social relationships. Obviously, the latter are more suitable as a cluster head user in D2D multicast cluster. What’s more, due to the differences in user’s relative distance and channel characteristics, the selection of cluster head affects the change of the worst signal to interference and noise ratio (${SINR}_{i}^{min}$), which in turn leads to different D2D transmission rates and system throughput. In summary, to better D2D multicast communication performance, selecting a suitable user as the cluster head is the key to data distribution.

In this paper, the channel model follows path fading and Rayleigh fading, each channel obeys one-mean Rayleigh fading independently, and assuming that the system knows the distance between all users, user’s status information, and history viewing information, etc. Therefore, the signal to interference and noise ratio of any two users can be calculated according to Equation (18), and then the channel quality matrix $\Gamma_{sinr}$ can be expressed. Likewise, the social relationship strength of any two users can be calculated according to Equation (16), and then the social relationship strength matrix **S** can be obtained. As a result, by traversing the user set $U_{C}$, we can calculate the value of D2D multicast utility metric when each user acts as cluster head according to Equation (22), further sorting out the cluster head that maximizes the multicast utility value.

By the above analysis, this paper proposes a cluster head selection algorithm to maximize utility value, in which the physical information of links and the social relationship information among users are considered. The details are shown in Algorithm 2.


**Algorithm 2. Utility Optimal Cluster Head Selection Algorithm**

**1.**

**Input:**

**2.**
users set $U_{C}$ of the cluster, distance matrix **D**, video request probability 
vector $P_{i}^{\mathrm{request}}$ of $u_{i}\in U_{C}$, initialize $u_{h}{=u}_{0}$, the max utility value $C_{\max}^{e}=0$, $u_{h}$

is the optimal cluster head
**3.**

**Output:**

**4.**
optimal cluster head selection scheme
**5.**

**Procedure Utility Optimal Cluster Head Selection Algorithm**

**6.**

**for**

$u_{i}\in U_{C}$

**do**

**7.**
    **for**
$u_{j}\in \left\{u_{j}{|u}_{j}\in U_{C}{,u}_{j}\neq u_{i}\right\}$
**do**
**8.**
         calculate the strength of social relations $s_{i,j}$ according to Equation (16), 
        and calculate the signal-to-interference noise ratio ${\mathrm{SINR}}_{i,j}$ according
        to Equation (18)
**9.**
    **end for**
**10.**

**end for**

**11.**

**for**

$u_{i}\in U_{C}$

**do**

**12.**
    calculate the utility metric $C_{i}^{e}$ according to Equation (22)
**13.**
    **if**
$C_{i}^{e}>C_{\max}^{e}$ then
**14.**
        Let $u_{h}=u_{i}$, $C_{\max}^{e}{=C}_{i}^{e}$

**15.**
    **end if**
**16.**

**end for**

**17.**
output the optimal cluster head $u_{h}$

**18.**

**End**


## 7. Simulation Analysis

### 7.1. Simulation Settings

In this section, the performance of the proposed user-information-aware D2D multicast file distribution mechanism is evaluated by using MATLAB. In the proposed file distribution mechanism, the video received by the service requesters is pushed to the potential service requesters so as to improve the throughput of the D2D multicast transmission system, further increasing the spectrum and energy efficiency. Therefore, in the experimental scheme, the value of D2D multicast utility metric and transmission rate in different communication environments is mainly compared. The relationships among the transmission performance, the number of potential service requesters ${|U}_{P}|$, the number of service requesters ${|U}_{R}|$, the number of files |F|, the weight of video’s popularity λ, and the willingness threshold to accept video push service $P_{\mathrm{th}}^{\mathrm{accept}}$ are studied in this section.

To fully verify the performance of the proposed clustering and cluster head selection mechanism, the proposed algorithm is simulated in different communication environments and compared with other schemes in the same communication environment.

● Scheme 1: Based on user information awareness

That is the proposed scheme in this paper, the nearby service requesters and the potential service requesters who satisfy a certain conditions constitute a D2D multicast cluster, and cluster head is selected by maximizing D2D multicast utility metric.

● Scheme 2: Based on social-aware method [19]

D2D multicast cluster consists only of the nearby requesters and the user who has the most stable social relationship with other users is selected as the cluster head.

● Scheme 3: Based on interference awareness [16]

The nearby service requesters constitute a D2D multicast cluster, the user who has the most stable links is selected as the cluster head.

● Scheme 4: Random cluster head selection

D2D multicast cluster is composed of the nearby service requesters, the cluster head is randomly selected in D2D multicast cluster.

Scheme 2 and Scheme 3 are typical solutions in the existing D2D multicast communication research, involving the user’s social information and channel quality information about users, respectively. The above two kinds of information are considered in the proposed user-information-aware D2D multicast file distribution mechanism. In fact, the basic principles of the first three schemes are the same, all of which can improve the system throughout by increasing the number of multicast receivers in the cluster, so Scheme 2 and Scheme 3 are selected as the comparative experimental scheme in this paper.

Considering a single-cell model, users are randomly distributed within the grid, and the locations of users follow the Homogeneous Poisson Point Process (HPPP) [32]. In addition, D2D communication users can reuse uplink resources with cellular users. The path fading model, Rayleigh fading model and Gaussian channel noise are considered in the channel model. The main experimental parameter settings are shown in Table 1. Notably, the parameters for any set of experiments, unless otherwise specified, are the default values in Table 1.

### 7.2. Effects of Various ${|U}_{P}|$ on Utility of the Proposed Scheme

**Figure 4:**
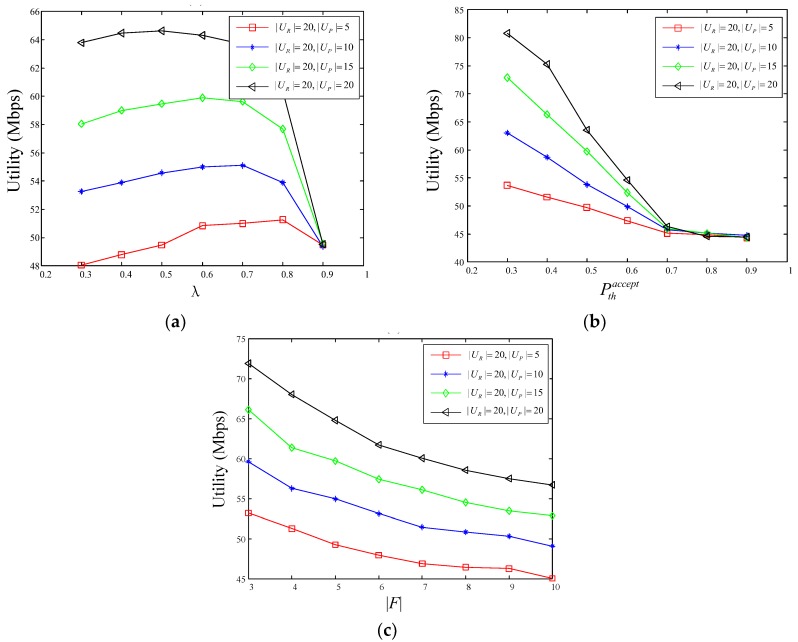
The relationship between utility and different communication environment: (**a**) Utility versus λ; (**b**) Utility versus $P_{\mathrm{th}}^{\mathrm{accept}}$; (**c**) Utility versus |F|.

Obviously, the number of the potential service requesters is a key factor influencing the performance of user-information-aware D2D multicast file distribution mechanism. To explore the relationship between the performance of the proposed mechanism and its own parameter settings, the performances in different communication environment of the proposed mechanism with different number of potential service requesters are studied in this section. As shown in Figure 4, by sequentially changing the weight of video’s popularity λ, the willingness threshold to accept video push service $P_{\mathrm{th}}^{\mathrm{accept}}$ and the number of files |F|, the performance of D2D multicast file distribution system with different number of potential service requesters shows a huge difference. In general, the utility value of a D2D multicast system is proportional to the number of potential servicing users ${|U}_{P}|$, where the value of D2D multicast utility metric reflects the average throughput of the D2D multicast file distribution system.

As shown in the Figure 4a, with the rise of the value of λ, the value of utility increases firstly and then decreases and the more potential service requesters, the faster the value of utility drops. That is because the increase in the value of λ makes that the video popularity $P_{l}^{\mathrm{pop}}$ has greater weight in calculating the user’s request probability, further reducing the difference of video request probability among users and increasing the intensity of social relationships, thereby the value of utility increases. Nevertheless, when the video popularity $P_{l}^{\mathrm{pop}}$ is a constant, the excessive value λ will cause the smaller absolute value of the willingness $P_{\mathrm{pi},l}^{\mathrm{accept}}$ so the number of potential service requesters who can meet the condition $P_{\mathrm{pi},l}^{\mathrm{accept}}>P_{\mathrm{th}}^{\mathrm{accept}}$ will decrease, leading to a decrease of utility value and the greater number of potential service requesters, the greater it decreases. As can be seen from Figure 4a, the utility value of the four curves can reach a maximum when the value of the λ belongs to the interval [0.4, 0.7], so we set the value of lambda in other experiments to 0.6.

As shown in Figure 4b, when the value of the user’s willingness threshold $P_{\mathrm{th}}^{\mathrm{accept}}$ continues to rise, the four curves all show a sharp downward trend until the willingness threshold is 0.7, and the greater number of potential service requesters, the greater the decline. That is because that, for any potential service requester in the D2D multicast cluster, the willingness to accept the pushed video file $f_{l}\in F$ must greater than the willingness threshold $P_{\mathrm{th}}^{\mathrm{accept}}$. If the value of $P_{\mathrm{th}}^{\mathrm{accept}}$ rises, the number of users meeting the conditions will decline, and the performance of utility will decrease dramatically. The simulations reveal that the value of the willingness $P_{\mathrm{pi},l}^{\mathrm{accept}}$ for most of the potential service requesters is less than 0.7. If the value of $P_{\mathrm{th}}^{\mathrm{accept}}$ is set too large, the system will not be able to better play its advantage because the number of potential service requesters satisfying the condition $P_{\mathrm{pi},l}^{\mathrm{accept}}>P_{\mathrm{th}}^{\mathrm{accept}}$ is reduced. If the value of $P_{\mathrm{th}}^{\mathrm{accept}}$ is set too small, many potential service requesters may be disturbed by the video push service, and then the QoE of users will be reduced, so in all experiments, the value of $P_{\mathrm{th}}^{\mathrm{accept}}$ defaults to 0.5.

The relationship between utility value and the number of videos |F| is shown in Figure 4c. According to Equation (6), as the number of videos increases, the popularity of individual video turns small, further reducing the willingness of potential service requesters to accept the pushed video, and thus the utility value become lower.

Through the above experiments and analysis, the D2D transmission performances of systems with different numbers of potential service requesters in different communication environments are clarified. In general, the parameter setting has a great influence on the system performance and the more potential requesters, the better the utility value for the system.

### 7.3. Effects of Various Parameters on Performance of All Four Schemes

#### 7.3.1. Effects of Various Parameters on Utility

In the above Section 7.2, we analyzed the proposed scheme’s performance through putting different number of potential requesters in different communication environments. In order to fully verify the superiority of the proposed scheme, in this subsection, the proposed scheme is compared with other schemes under different communication environment.

**Figure 5:**
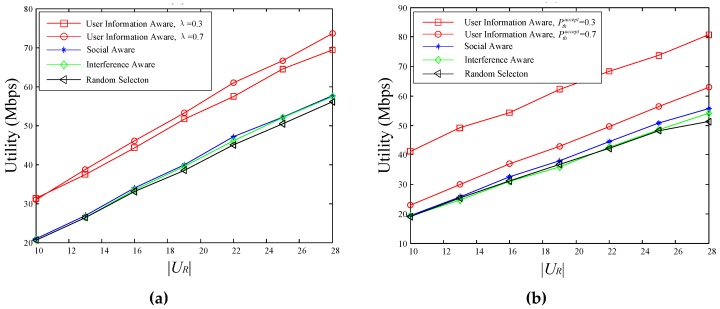
The relationships between utility and ${|U}_{R}|$: (**a**) $P_{\mathrm{th}}^{\mathrm{accept}}$ = 0.5; (**b**) λ = 0.6.

The relationships between utility value and the number of service requesters are shown in Figure 5. In Figure 5a, the value of $P_{\mathrm{th}}^{\mathrm{accept}}$ is set to 0.5, the first curve and the second curve represent the performance of the proposed scheme when the value of λ is set to 0.3 and 0.7, respectively. And in Figure 5b, the value of λ is set to 0.6, the first curve and the second curve represent the performance of the proposed scheme when the value of $P_{\mathrm{th}}^{\mathrm{accept}}$ is set to 0.3 and 0.7, respectively.

As shown in Figure 5, with the increase of service requesters, the utility value of four schemes also rises with varied degrees, and the rise of the proposed scheme is slightly larger than the other three schemes. As the process of ${|U}_{R}|$ increases, the performance of the proposed scheme is obviously superior to the other schemes. Scheme 2 with the consideration of social information and scheme 3 with the consideration of interference information have similar performances, and the performance of scheme 4 by selecting cluster head randomly is the worst.

For the proposed scheme, when λ is set to 0.7, its performance is better than when λ is set to 0.3. This is because the larger the λ, the greater the weight of the video popularity in the calculation of the user’s request probability. This will increase the similarity of the request probability vectors of different users, and thus the social relationship strength value will become larger. Further, the utility value becomes larger. The choice of threshold $P_{\mathrm{th}}^{\mathrm{accept}}$ is an important factor affecting the number of the potential requesters who meet the condition $P_{\mathrm{pi},l}^{\mathrm{accept}}>P_{\mathrm{th}}^{\mathrm{accept}}$. As shown in Figure 5b, when $P_{\mathrm{th}}^{\mathrm{accept}}$ is set to 0.7, the performance of the proposed scheme is much lower than when $P_{\mathrm{th}}^{\mathrm{accept}}$ is set to 0.3. It is gratifying that the performance of the proposed scheme under different parameter settings is always better than the comparison schemes.

**Figure 6:**
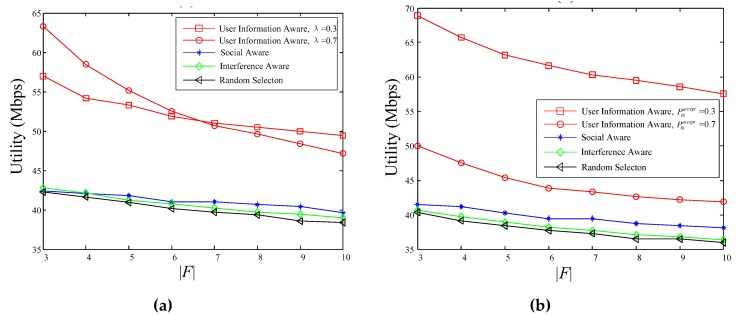
The relationship between utility and |F|: (**a**) $P_{\mathrm{th}}^{\mathrm{accept}}$ = 0.5; (**b**) λ = 0.6.

As noted above, when the number of video files |F| increases, the popularity of individual video shows decrease, making that the weight of user’s interest in the calculation of video request probability increases, further resulting in a decrease in the similarity of the video request probability between users, in turn the strength of social relationships between users decreases. What’s more, in the proposed user’s information awareness scheme, the increase of |F| can cause low probability of potential requesters to accept the pushed videos, and then the number of users who meet the condition $P_{\mathrm{pi},l}^{\mathrm{accept}}>P_{\mathrm{th}}^{\mathrm{accept}}$ also decreases. Therefore, Figure 6 shows that the utility value of all four schemes decreases dramatically as the number of video files increases. Due to the decrease of both the intensity of social relations and the number of potential servicing users, the decrease speed of the proposed scheme is faster than the other three schemes, but the utility value of the proposed scheme is still greater than the other three schemes. For the same reason mentioned above, the performance of the proposed scheme differs under different parameter settings.

To summarize, in the proposed user-information-aware D2D multicast file distribution mechanism, the video is pushed to potential service requesters when distributing video for service requesters. Therefore, the number of the D2D multicast receivers is far greater than the other three schemes, and it is easier to select a more suitable cluster head, resulting in a better utility value compared with the other three schemes. In other words, the efficiency of spectrum and energy are improved greatly. What’s more, the robustness of the proposed scheme is also proved by comparing the performance of the proposed scheme under different parameter settings.

#### 7.3.2. Effects of Various Parameters on Transmission Rate

By predicting the potential requester’s willingness, the proposed user-information-aware D2D multicast file distribution mechanism pushes the video to potential requesters whose willingness greater than willingness threshold. The throughput and resource utilization efficiency are improved greatly by increasing the number of D2D multicast receivers. The utility values of all four schemes have studied in the above subsection, and the D2D transmission rate of all four schemes are studied in this subsection.

**Figure 7:**
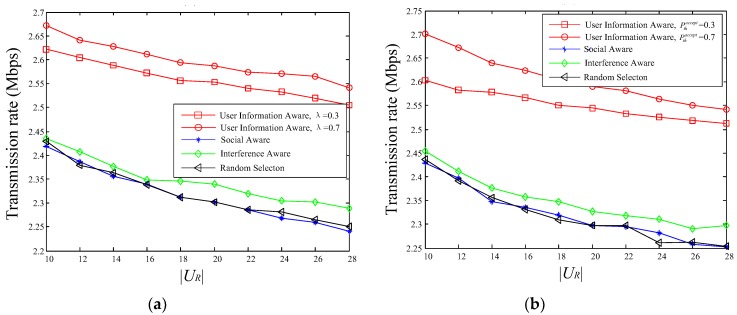
The relationships between transmission rate and ${|U}_{R}|$: (**a**) $P_{\mathrm{th}}^{\mathrm{accept}}$ = 0.5; (**b**) λ = 0.6.

The relationships between the D2D transmission rate and the number of service requesters ${|U}_{R}|$ are shown in Figure 7, and we can easily find that the transmission rate of the proposed scheme is greater than the other three schemes. With the number of service requesters increases, the performance of the user-information-aware scheme is still better than other three schemes, although the transmission rate of all four schemes has declined to varying degrees. The interference-aware scheme is better than social-aware scheme and the Random cluster-head selection scheme. What’s more, the decline of user-information-aware scheme slower than other three schemes, followed by the interference-aware scheme. Compared with the social-aware scheme and the random cluster-head selection scheme, the channel quality information between users is considered in the interference-aware scheme, which makes the transmission rate of the interference-aware scheme is better than the above two schemes. Although the purpose of the proposed scheme is to maximize the utility value, the transmission rate is also better than the other schemes. It can be seen from Equation (22) that this scheme considers the channel quality information and social relationship information at the same time, and owns more members in the D2D multicast cluster to facilitate the selection of suitable cluster head. 

Since the values of λ and $P_{\mathrm{th}}^{\mathrm{accept}}$ affect the number of potential service requesters satisfying the condition $P_{\mathrm{pi},l}^{\mathrm{accept}}>P_{\mathrm{th}}^{\mathrm{accept}}$, the selection of cluster head is affected. Therefore, the performance of the proposed scheme differs under different parameter settings, but it is still superior to the comparison schemes.

### 7.4. Experiment Conclusion

Based on the above experiments and analysis, the performance of the proposed mechanism has been fully verified. Owing to the advantages of large number of D2D multicast receiving users, the utility value of the proposed scheme has greatly improved. And compared with other schemes, even though changing the communication environments, the transmission rate is also better. The efficiency of spectrum and energy has improved dramatically. More importantly, the performance advantage can be more significant as the number of potential requesters increases.

## 8. Conclusions

D2D communication, as a promising technology to solve the shortage of wireless spectrum resources, can provide more high-quality multimedia services for users. By D2D multicast file distribution technology, the redundant transmissions of multimedia files can be reduced, then dramatically improving the efficiency of spectrum and energy. So, substantial research effort has been undertaken to improve the performance of D2D multicast communication. However, the distribution of user’s service requests in the time domain is not fully considered in the existing design of D2D multicast communication system. There are a large number of users who have the same service demands with different service request times, which cause repeat transmission for same content, and the efficiency of spectrum and energy can be further improved. In this paper, the willingness of potential service requesters to accept the video push service is predicted firstly, and then the video is pushed to potential service requesters when distributing video for service requesters. The user-information-aware D2D multicast file distribution mechanism is proposed and the efficiency of spectrum and energy are improved dramatically by increasing the number of multicast receivers. To guarantee the QoE of potential service requesters, the users’ interests and status information of user’s device are fully taken into account in the calculation of the willingness of potential requesters. With the consideration of the social relationships and the channel quality information about users, the D2D multicast utility metric is proposed, and then a cluster head selection scheme is developed to maximize the utility value. The simulation results show that the file distribution mechanism proposed in this paper can greatly improve the system’s throughput, thus improving the efficiency of spectrum and energy, and the transmission rate also has an excellent performance.

## Figures and Tables

**Table 1:** Simulation parameters.

Parameters	Values
The maximum distance (D2D)	60 m
Channel bandwidth *B*	180 KHz
Path loss exponent α	4
Noise spectral density	−174 dBm/Hz
The circuit loss constant $E_{\mathrm{elec}}$	50 nJ/bit
Cluster head transmit power $P_{h}$	23 dBm
Cellular user transmit power $P_{c}$	30 dBm
The willingness threshold $P_{\mathrm{th}}^{\mathrm{accept}}$	0.5
The number of service requesters ${|U}_{R}|$	20
The number of potential service requesters ${|U}_{P}|$	10
The number of videos |F|	5
The weight of video popularity λ	0.6
